# Prevention of Decline in Cognition after Stroke Trial (PODCAST): a study protocol for a factorial randomised controlled trial of intensive versus guideline lowering of blood pressure and lipids

**DOI:** 10.1186/1745-6215-14-401

**Published:** 2013-11-22

**Authors:** Daniel J Blackburn, Kailash Krishnan, Lydia Fox, Clive Ballard, Alistair Burns, Gary A Ford, Jonathan Mant, Peter Passmore, Stuart Pocock, John Reckless, Nikola Sprigg, Rob Stewart, Joanna Wardlaw, Philip MW Bath

**Affiliations:** 1 Sheffield Institute for Translational Neuroscience, University of Sheffield, 385A Glossop Road, Sheffield S10 2HQ, UK; 2 Stroke Trials Unit, Division of Clinical Neuroscience, University of Nottingham, City Hospital campus, Nottingham NG5 1PB, UK; 3 Wolfson Centre for Age-Related Diseases, Wolfson Wing, Hodgkin Building, King’s College London, Guy’s Campus, London SE1 1UL, UK; 4 Faculty of Medical and Human Sciences, Institute of Brain, Behavior and Mental Health, University of Manchester, Grafton Street, Manchester M13 9NT, UK; 5 Level 6, Leazes Wing, Royal Victoria Infirmary, Newcastle NE14 LP5, UK; 6 General Practice & Primary Care Research Unit, University of Cambridge, Addenbrooke’s Hospital, Forvie Site, Cambridge CB2 0SR, UK; 7 Institute of Clinical Sciences, Queens University, Belfast, Royal Victoria Hospital, Block B, Belfast BT12 6BA, UK; 8 Department of Medical Statistics, London School of Hygiene and Tropical Medicine, Keppel Street, London WC1E 7HT, UK; 9 Department of Endocrinology, Royal United Hospital, Combe Park, Bath BA1 3NG, UK; 10 Section of Epidemiology (Box 60), Institute of Psychiatry (King's College London), De Crespigny Park, London SE5 8AF, UK; 11 Division of Clinical Neurosciences, Western General Hospital, Crewe Rd, Edinburgh EH4 2XU, UK

**Keywords:** Stroke, Post-stroke cognitive impairment, Post-stroke dementia, Blood pressure lowering, Lipid lowering

## Abstract

**Background:**

Stroke is a common cause of cognitive impairment and dementia. However, effective strategies for reducing the risk of post-stroke dementia remain undefined. Potential strategies include intensive lowering of blood pressure and/or lipids.

**Methods/Design:**

Design: multi-centre prospective randomised open-label blinded-endpoint controlled partial-factorial phase IV trial in secondary and primary care.

Participants: 100 participants from 30 UK Stroke Research Network sites who are post- ischemic stroke or intracerebral haemorrhage by three to seven months.

Interventions - all patients (1:1): intensive versus guideline blood pressure lowering (target systolic < 125 mmHg versus < 140 mmHg).

Interventions - ischemic stroke (1:1): intensive versus guideline lipid lowering (target low density lipoprotein-cholesterol (LDL-c) < 1.4 mmol/l versus < 3 mmol/l).

Hypotheses: does ‘intensive’ blood pressure lowering therapy and/or ‘intensive’ lipid control reduce cognitive decline and dementia in people with ischemic stroke; and does ‘intensive’ blood pressure lowering therapy reduce cognitive decline and dementia in patients with hemorrhagic stroke.

Primary outcome: Addenbrooke’s Cognitive Examination-Revised.

Secondary outcomes: feasibility of recruitment and retention of participants, tolerability and safety of the interventions, achieving and maintaining the blood pressure and lipid targets, maintaining differences in systolic blood pressure (> 10 mmHg) and low density lipoprotein-cholesterol (> 1 mmol/l) between the treatment groups, and performing clinic and telephone follow-up of cognition measures.

Randomisation: using stratification, minimization and simple randomization.

Blinding: participants receive open-label management. Cognition is assessed both unblinded (in clinic) and blinded (by telephone) to treatment. Adjudication of events (dementia, vascular, serious adverse events) is blinded to management.

**Discussion:**

The PODCAST trial is ongoing with 78 patients recruited to date from 22 sites. Outcomes of cognitive impairment and dementia are accruing.

**Trial registration:**

ISRCTN85562386

## Background

### Post-stroke cognitive impairment

Post-stroke cognitive impairment is common, ranging from 17 to 92%, [1,2] and is associated with increased mortality and decreased quality of life [3-5]. Nevertheless, cognitive impairment may improve or deteriorate following a stroke [6]. Risk factors for cognitive decline include executive dysfunction, white matter hyperintensities (WMH), ApoE e4 status [7] and atrophy of crucial brain areas [8].

Many potential interventions for preventing cognitive decline have been proposed, including blood pressure (BP) and lipid lowering, antiplatelet agents, anti-oxidant vitamins, and cholinesterase inhibitors. Of these, lowering BP and blood lipids are priorities for testing.

### Blood pressure lowering

Lowering BP post-stroke is highly effective in reducing recurrent and other vascular events, as shown in individual trials (such as Post-stroke Antihypertensive Treatment Study (PATS, n = 5,665) and Perindopril protection against recurrent stroke study (PROGRESS, n = 6,105) [9,10]) and a meta-analysis of them [11]. However, the effect on cognitive function of lowering BP is far less clear.

Longitudinal studies have shown that premorbid high systolic blood pressure (SBP) and diastolic blood pressure (DBP) are associated with WMH and an increased risk of Alzheimer’s disease (AD) and vascular dementia [12-14]. Although no trials have been expressly designed to test the effect of lowering BP on subsequent cognition post-stroke, several have included cognition as a secondary outcome measure. Whilst potential benefit was seen in the PROGRESS study [15], none was found in the Prevention Regimen for Effectively Avoiding Second Strokes trial (PRoFESS, n = 20,332) [16]. Similar mixed results have been seen in trials of BP lowering in non-stroke populations, for example, the Hypertension in the Very Elderly Trial (HYVET, n = 3,845) and Systolic Hypertension in Europe (Syst-Eur, n = 4,695) [17,18]. In a meta-analysis involving both stroke and non-stroke patients, lowering BP was associated with less cognitive decline, and a trend to less dementia [19]; meta-regression suggested that the degree of reduction in cognition was related to the magnitude of BP lowering.

The Secondary Prevention of Small Subcortical Strokes (SPS3, n = 3,020) factorial trial of intensive versus guideline BP lowering, and aspirin/clopidogrel versus aspirin [20], in patients with MRI-proven lacunar stroke will be presenting the effects of intensive BP lowering on cognition [21] in 2014. The ongoing PRESsure in established cERebral small VEssel disease (PRESERVE, n = 422) trial is also investigating the effect of lowering BP in patients with established cerebral small vessel disease (http://www.controlled-trials.com/ISRCTN37694103, downloaded 20 June 2013).

### Lipid lowering

The majority of information on lipid lowering and cognition relates to statins rather than older interventions such as fibrates, nicotinic acid derivatives or resins. Statins have pleiotropic effects that include lowering cholesterol (specifically low density lipoprotein-cholesterol (LDL-c)) and reducing platelet activity, inflammation and the release of cytokines and acute phase reactants [22,23]. These effects might limit the progression of Alzheimer’s pathology from an asymptomatic state to symptomatic, or deterioration after stroke [24]. Although statins are one of the most widely prescribed drugs with clear health benefits in reducing vascular events, including stroke [25-27], and death, there is little direct evidence that lipid lowering prevents cognitive decline in either people with normal cognition or patients with cognitive impairment.

The Heart Protection Study (HPS, n = 20,536) found significant reductions in coronary artery and cerebrovascular events with simvastatin [28] but there was no difference in cognition on treatment (baseline measures were not taken so change could not be assessed), assessed using the Telephone Interview for Cognitive Status (TICS), even when sub-groups of older patients, and those with prior stroke, were analysed. Similarly, the Pravastatin in elderly individuals at risk of vascular disease (PROSPER, n = 5,804) trial in people aged 70 to 82 with vascular risk factors reported no effect on cognition (measured using Mini Mental State Examination (MMSE), Stroop and a series of psychometric tests) [29]. A meta-analysis of three trials found a non-significant trend to higher MMSE scores in patients with AD who were randomised to statin treatment (atorvastatin, simvastatin) [19].

Thus, there is no clear evidence that statins reduce the risk of cognitive decline or dementia but this has not been formally examined in a high-risk population. In contrast, there has been some concern that statins are associated with reversible cognitive impairment [30], either due to an idiosyncratic response to statins or an underlying mitochondrial dysfunction.

## Methods/design

### Purpose

To develop interventions to prevent cognitive decline and dementia after stroke.

### Primary objectives

#### Start-up phase

To determine the feasibility of recruiting and retaining patients, and identify any barriers to achieving BP and lipid targets.

#### Main phase

To determine if ‘intensive’ blood pressure lowering therapy, and/or ‘intensive’ lipid lowering therapy, after stroke reduces cognitive decline and dementia.

### Secondary objectives

#### Start-up phase

To determine the feasibility of recruiting and retaining sites, reaching and maintaining target BP and lipid levels, performing cognitive assessment in clinic and by telephone, and the tolerability and safety of the management strategies.

#### Main phase

To determine if ‘intensive’ blood pressure lowering therapy, and/or ‘intensive’ lipid lowering therapy, after stroke reduces poor quality of life, poor function, depression, stroke recurrence, vascular events, and death.

### Aims

#### Start-up phase

This is assessing the:

•Ability to deliver the protocol

•Ability to recruit 30 recruiting sites

•Ability to recruit and retain 600 participants

•Ability to achieve and maintain differences in systolic BP ≥ 10 mmHg and LDL-c ≥ 1 mmol/l between the ‘intensive’ and ‘guideline’ treatment groups

•Ability to perform clinic and telephone follow-up of outcome measures

•Sensitivity of the Addenbrooke’s Cognitive Examination-R (ACE-R) and other cognitive measures to change over time

•Tolerability and safety of the intervention

#### Main phase

A main phase was planned to assess the safety and efficacy of intensive versus guideline BP and lipid management in preventing cognitive decline. A total of 3,400 patients (start-up 600, main 2,800) post-stroke were planned. However, the main phase was cancelled 24 months into the pilot phase because of a failure to achieve a sufficiently high recruitment rate.

### Design

PODCAST is a multi-centre prospective randomised open-label blinded-endpoint controlled partial-factorial phase IV trial. The study is conducted according to the principles of the Declaration of Helsinki and ‘International Conference on Harmonisation of Good Clinical Practice’. Study approval by national (UK, approval 09/H0403/71, date 12 November 2009) and local research ethics committees (all centres) has been obtained. As a management trial, the study does not fall under the remit of the UK Medicines and Healthcare Products Regulatory Authority (as confirmed by them). The management of personal data adheres to the UK Data Protection Act 1998. The UK National Institutes Health Research Stroke Research Network supports the trial through screening and recruitment of patients (23 September 2009).

### Patient population

Participants are recruited from hospital-based stroke services, and are consented for a face-to face assessment of cognition (telephone-Mini Mental Status Examination, t-MMSE) and function (modified Rankin Scale, mRS) at 8 to 26 weeks after the stroke. If the participant is eligible and interested after the initial assessment, fasting lipids, glucose, urea and electrolytes, and HbA1c are tested.

#### Inclusion criteria

1. Age > 70 years and t-MMSE > 16 (maximum score 22); or age > 60 years and t-MMSE 17 to 20

2. Functionally independent (mRS 0 to 2).

3. Ischemic stroke (IS, any Oxfordshire Community Stroke Project or Trial of Org10172 in Acute Stroke Treatment type [31,32]) or spontaneous intracerebral haemorrhage.

4. Three to seven months post-event (to allow cognitive, neurological, BP and lipid stabilisation [33], but avoid attrition).

5. Systolic BP 125 to 170 mmHg.

6. Total cholesterol (TC) 3 to 8 mmol/l.

7. Presence of an informant (ideally two): partner, sibling, child, friend (for Informant Questionnaire on Cognitive Decline in the Elderly, IQCODE [34]).

8. Capacity and willingness to give consent.

#### Exclusion criteria

1. Participants not meeting inclusion criteria.

2. Subarachnoid haemorrhage.

3. Secondary intracranial haemorrhage (trauma, arterio-venous malformation, cavernoma).

4. No CT/MR brain scan within ten days of index stroke.

5. Inability to give consent or do study measures, for example, severe dysphasia, weakness of dominant arm.

6. Profound deafness.

7. Severe hypertension (systolic BP > 170 mmHg).

8. Definite need for ‘intensive’ BP control.

9. Severe hypercholesterolemia (TC > 8 mmol/l).

10. Definite need for, or demonstrated intolerance of, ‘high intensity’ statin.

11. Definite need for a cholinesterase inhibitor for dementia.

12. Familial stroke associated with dementia, for example, CADASIL.

13. Chronic renal failure: eGFR < 45 (or eGFR < 37 in people of African/Afro-Caribbean origin).

14. Liver disease, ALT > three times upper limit of normal, using local laboratories ranges.

15. Ongoing participation in trials involving drug and/or devices. Participants already in another trial may be recruited to PODCAST, provided that participation in the other trial is complete prior to PODCAST randomisation.

16. Any serious medical co-morbidity (for example, active malignancy) such that the life expectancy is < 24 months.

17. Clinically unstable at the time of enrollment.

18. Dementia.

### Informed consent

All participants must have capacity, and be willing and able to provide written informed consent. Participants are screened for potential recruitment during their initial presentation to the hospital stroke services, and are given an information sheet explaining the study.

#### Screening consent

Informed consent for formal screening is taken in hospital for conducting the following assessments, 8 to 26 weeks after their stroke:

•Assessment of cognition - t-MMSE

•Assessment of function - mRS

•Blood test - fasting lipids, glucose, urea and electrolytes, HbA1c

The availability of an informant (partner, sibling, child or friend), ideally with a backup, is key. Informants provide information on the participant’s prior cognitive state and decline (via the IQCODE).

Both the patient and informant are then given information sheets to take away and review.

#### Full consent

Providing the patient fulfils the inclusion–exclusion criteria at the screening visit, full consent is taken at the baseline visit. This includes an assessment of capacity by telling the patient about the trial and then asking them to answer questions based on this information:

•What condition? Stroke

•What is the trial trying to prevent? Dementia

•What are the interventions? Intensive BP and/or lipid lowering

Following any questions about the trial, written informed consent of both the patient and informant is then performed. Patients may also give consent for two sub-studies:

•Ambulatory BP monitoring

•On-treatment CT scan

Part of the consent process involves both the patient and informant agreeing to the latter assuming the right of proxy consent if the patient loses capacity during the trial.

### Randomisation

Eligible and consenting participants are randomised centrally using a secure internet site in real-time: https://www.nottingham.ac.uk/~nszwww/podcast/podcasttrialdb/podcast_login.php.

The process of randomisation includes stratification, minimisation and simple randomisation, based on information gathered by the local recruiting investigators. Stratification and minimisation allow for improved matching at baseline: stratification allows variable categories to be treated as nested trials in their own right; minimisation increases statistical power [35]. Simple randomisations reduce predictability. The minimisation variables will be used for adjustment of the primary and secondary analyses.

#### Stratification

•Stroke type (IS, spontaneous ICH)

Patients with IS are randomised to both BP lowering (intensive versus guideline) and lipid lowering (intensive versus guideline) strategies.

Patients with ICH are randomised to BP lowering (intensive versus guideline) strategy only.

#### Minimisation (on key prognostic/logistical variables)

•Age (< 70/> 70 years)

•Sex (female, male)

•Dysphasia (no, yes)

•Cognition, ACE-R (≥ 85/< 85)

•Systolic BP (< 150 / > 150 mmHg)

•Total cholesterol (< 4.0/≥ 4.0 mmol/L)

•Function/dependency, mRS (0/≥ 1)

•Brain region (subcortex/cortex)

•Evidence of periventricular white matter lucency (no, yes)

•Time since index stroke (< 140/> 140 days)

•Number of antihypertensive drugs (< 2/≥ 2)

•Already on a statin (yes, no)

#### Simple randomisation

On 5% of patients at time of minimisation.

#### Randomisation groups

Study participants are randomized to:

•Intensive versus guideline BP lowering - all participants

•Intensive versus guideline lipid lowering - ischaemic stroke only

As a result, patients can be randomised to one of six groups:

1. Intensive BP lowering and intensive lipid lowering (ischaemic stroke only)

2. Intensive BP lowering and guideline lipid lowering (ischaemic stroke only)

3. Intensive BP lowering only (ICH only)

4. Guideline BP lowering and intensive lipid lowering (ischaemic stroke only)

5. Guideline BP lowering and guideline lipid lowering (ischaemic stroke only)

6. Guideline BP lowering only (ICH only)

Assuming that approximately 10% of patients will be enrolled with ICH, the distribution of patients between the six treatment groups will, for every 100 patients, approximate to 22.5% in each of the four groups of ischaemic stroke patients (intensive versus guideline BP; intensive versus guideline lipid) and 5% in each group of patients with ICH (intensive versus guideline BP).

### Interventions

The trial is assessing management strategies (‘intensive’ versus ‘guideline’) rather than particular drugs. All participants receive standard lifestyle advice and rehabilitation (as per NICECG 68, 2008 [36]) including: diet, exercise, smoking advice, rehabilitation, psychological assessment and therapy, modification of all risk factors and other relevant interventions.

#### Guideline management

Participants randomised to the guideline groups are managed by their general practitioner (GP) who follows national/international guidelines and local practice.

#### Guideline BP lowering

It is expected that GPs will aim for a systolic BP < 140 mmHg.

#### Guideline lipid lowering

It is expected that GPs will aim for a LDL-c < 3 mmol/l (or TC < 5 mmol/l).

#### Intensive management

Participants in the intensive group are managed by the local hospital stroke research team and medications initiated by the local investigator and continued by the GP. The trial does not stipulate specific drugs but gives examples of drugs (and relevant doses) to use from the different drug classes. Guidance on which drugs to start and add, how to titrate, and how to manage participants with various contra-indications to medications, are included in algorithms; these are updated to include new information as relevant.

Intensive BP and lipid management strategies may be attenuated or stopped if the patient or their informant withdraws consent, for safety, or if unacceptable adverse events develop. If the participant wishes to withdraw from treatment, they are requested to permit primary outcome data to be collected, ideally at the end of the follow-up period.

#### Intensive BP lowering

Two targets are required for intensive BP lowering:

•Systolic BP < 125 mmHg

•Difference in systolic BP between intensive and guideline groups > 10 mmHg

Additional guidance on salt and alcohol restriction, and weight reduction is given. The intensive BP treatment algorithm is based on NICE guidelines relating to stroke (CG68 2008 [36]), hypertension (CG127 2011) and type 2 diabetes (CG66 2006, partially updated by CG87 [37]). The algorithm is only a guide and investigators may choose other medications depending on local policy and practice as long as they fit with the overall design of the trial, that is, to achieve intensive BP lowering.

Suitable drug classes and example drugs are:

•‘A’ = angiotensin converting enzyme inhibitor (ACE-I, for example, perindopril 2 to 8 mg daily (od), ramipril 1.25 to 5 mg twice daily (bd)) or angiotensin receptor antagonist (ARA, for example, losartan 25 to 100 mg od, candesartan 8 to 32 mg od)

•‘B’ = beta (β)-receptor antagonist (for example, atenolol 25 to 100 mg od, bisoprolol 5 to 20 mg od)

•‘C’ = calcium channel blocker (for example, amlodipine 5 to 10 mg od, nifedipine LA 30–60 mg od, verapamil SR 120 to 240 mg od)

•‘D’ = diuretic (for example, bendroflumethiazide 2.5 mg od, hydrochlorothiazide 12.5 mg od, indapamide 2.5 mg od)

•‘K’ = potassium-sparing diuretics (for example, spironolactone 12.5 to 100 mg od, amiloride 5 to 20 mg od)

•‘Z’ = alpha (α)-receptor antagonists (for example, doxazosin 4 to 16 mg od)

•‘M’ = centrally acting drugs (for example, moxonidine 200 to 600 μg daily in divided doses)

In the absence of contraindications, participants should be started on either:

•An ‘A’ drug, with subsequent addition of a ‘C’ then ‘D’ drug (as required)

•A ‘C’ drug, with subsequent addition of an ‘A’ then ‘D’ drug (as required)

If additional treatment is needed to reach target, fourth-line and additional options include:

•Add a K or Z drug, then the other

•Add an ‘M’ drug

#### Intensive lipid lowering

Two targets are required for intensive lipid lowering:

•Calculated LDL-c < 1.4 mmol/l

•Difference in LDL-c between intensive and guideline groups > 1.0 mmol/l

If LDL-c cannot be calculated (for example, due to an elevated triglyceride level), targets for TC are used instead:

•TC < 3.1 mmol/l

•Difference in TC between intensive and guideline groups > 1.0 mmol/l

Drug therapy for the intensive lipid arm will typically comprise:

•A third-generation statin (for example, atorvastatin 80 mg od) [38]

•Then add ezetimibe (10 mg od)

•Then add a resin

Additional guidance on the use of plant stanols/sterols as part of meals, and weight reduction is given. The algorithm for intensive lipid reduction builds on NICE guidelines (CG67, 2008 [39], ezetimibe [40]). Again, the algorithm is only a guide and investigators may choose other treatment strategies depending on local policy and practice as long as they fit with the overall design of the trial, that is, to achieve intensive lipid lowering.

#### Standard care

Participants receive standard evidence-based care on top of the interventions, including (as appropriate):

•IS: anticoagulation (cardioembolic stroke), antiplatelets (other IS), carotid endarterectomy

### Blood pressure and lipid measurements

#### Blood pressure (BP)

BP measurements are performed using a validated automated BP monitor, for example, Omron 705CP or 705CP II. These devices have been validated by the British Hypertension Society [41], and were used in the positive ASCOT hypertension mega-trial involving 20,000 patients [42]. Baseline and follow-up systolic and diastolic BP and heart rate (HR) readings are taken by trained staff in the non-paretic arm with the participant sitting (three readings) and then standing (one reading).

#### Ambulatory BP monitoring (ABPM)

In centers with ABPM equipment (for example, SpaceLabs 90207), participants have 24 hour ABPM performed at baseline and than on treatment every six months. Twenty-four hour, day-time (07.00 to 23.00 thrice hourly) and night-time (23.00 to 07.00 hourly) ABPM data are recorded. From these, a number of measures are calculated:

•Mean SBP, DBP and HR for each time interval

•Peak SBP and HR profile over 24 hours

•BP and HR variation as standard deviation and coefficient of variation (= SD/Mean)

#### Lipid measurement

Fasting lipids are measured at an accredited clinical biochemistry laboratory. Fasting should be performed overnight and measurements made at least one month after the last change in lipid lowering therapy. Lipid measurements utilize standard techniques and comprise:

•TC

•Triglyceride (TG)

•HDL cholesterol (HDL-c)

•LDL-c (calculated)

### Outcome measure

#### Screening

An abbreviated form of the t-MMSE is used to screen patients so that those with dementia are excluded.

#### Primary outcome

The primary outcome is the Addenbrooke’s Cognitive Examination-Revised (ACE-R), which includes the MMSE. The ACE-R is measured at baseline and at each six-month research clinic visit.

#### Secondary outcomes

These are assessed at baseline and at each six-month research clinic visit.

•Cognitive outcomes, participant: MoCA, TICS, Stroop and trail-making A and B

•Cognitive assessment, informant: IQCODE

•Cognitive impairment (ACE-R < 89)

•Cognitive decline (reduction in ACE-R by ≥ 10, or ACE-R < 89)

•Dementia (DSM IV)

•Quality of life: Euro-Qol (EQ-5D and EQ-VAS). A health utility status will be calculated from the EQ-5D using the UK version of the time trade-off algorithm

•Mood: Zung Depression rating Scale (ZDS, short form)

•Function: modified Rankin Scale (mRS), Barthel Index (BI)

•Health resource utilisation: face-to-face survey with participant and carer

•Vascular event: stroke recurrence (by type), myocardial infarction (MI), peripheral arterial disease (PAD)

•Serious adverse events (SAE)

•Disposition: home, home with carer, residential home, nursing home, hospital, death

•Haemodynamics: blood pressure, heart rate

•Blood: fasting lipids (TC, TG, HDL-c, LDL-c)

•Blood: Urea and electrolytes, glucose, HbA1c

A head CT/MR scan is performed once participants have been in the trial at least twelve months (six months minimum). Comparison of this with the index stroke CT scan will allow changes to be identified: new stroke lesions, white matter disease, atrophy.

Dementia, vascular events, and brains scans are adjudicated by experts blinded to treatment assignment. All patients are registered with the Office for National Statistics to identify death and its certified cause.

### Sample size calculation

#### Start-up phase

Recruitment of 600 participants (300 per BP group, approximately, 270 per statin group) will be sufficient to demonstrate adequacy in recruitment of sites and participants, whether sufficient on-treatment differences in BP and lipids can be obtained and maintained, and whether cognition can be assessed satisfactorily. No formal sample size calculation is relevant to this part of the trial.

#### Main phase

Using the ACE-R, expanded to include death, as the primary outcome, the whole trial (start-up plus main phases) will need a sample size of 3,400 (1,700 per BP group) post-stroke participants, assuming:

•Significance, α = 5%

•Power (1-β) = 90%

•Rate of cognitive impairment or death in guideline BP group = 25% at five years (main trial, average length of follow-up four years) [34]

•Rate of cognitive impairment or death in ‘intensive’ BP group = 20%, that is, absolute risk reduction (ARR) = 5% (number-needed-to-treat = 20), relative risk reduction (RRR) = 20%

•Losses to follow-up = 3%

Hence, 765 participants (0.225 × 3,400) are anticipated to develop cognitive impairment or die. The sample size allows a smaller but clinically worthwhile decline in cognitive decline to be identified with 80% power, that is, ARR = 4.5% (RRR 18%). Since there are less existing data on the effect of cholesterol lowering on cognition, the statin factor will assume the same RRR (20%) but have less power (approximately 86%) since it will only involve participants with ischemic stroke (approximately 3,060).

Changing from a binary to ordinal analysis of cognitive outcomes may allow for a reduction in sample size of up to 30%, as seen in the ‘Optimizing Analysis of Stroke Trials’ (OAST) collaboration for functional outcome after stroke [43]. Providing ordinal analysis appears to be more efficient than binary analysis for cognition data, the trial will be re-sized according to the method of Whitehead [44]. Analyses will be adjusted for the covariates since this approach increases statistical power [45] and is recommended by the European Agency for the Evaluation of Medicinal Products (EMEA) Ref CPMP/EWP/560/98). Any such decision to change will be performed prior to database lock, blinded to treatment, and defined explicitly in the Statistical Analysis Plan.

### Statistical analysis

#### Feasibility of start-up phase

The feasibility criteria listed in section 2.4.1 are reviewed during the trial. A review at 24 months found that there was no chance of recruiting 600 patients during the internal pilot and that, therefore, the planned main phase should be cancelled (see section 4).

#### Comparisons between treatment groups

Outcomes will be compared between the treatment groups by intention-to-treat (ITT):

•Intensive versus guideline BP lowering

•Intensive versus guideline lipid lowering

Analyses will be adjusted for baseline values and stroke type, age, sex, SBP, TC, and time from stroke to randomization. Continuous covariates (age, SBP, TC, time) will be used with their raw data, that is, not dichotomized. (The full set of stratification and minimization variables listed in section Randomisation will not be used for adjustment because of the limited anticipated sample size of approximately 100).

#### Missing data, and death

Missing data will not be imputed. Participants who die will be assigned discrete values for outcome measures with a value worse than any living value (as is standard for mRS, BI). This avoids giving death the same value as the worst possible outcome when alive (best to worst) or, worse, excluding patients who die (since many dementia trials have been confounded by losses to death). Hence, patients who die will be included in all analyses. The EQ-5D Health Utility State (HUS) gives death a score of 0.

•Addenbrooke’s Cognitive Examination-Revised (ACE-R), 100 to 0 with death = −1

•Mini-Mental State Examination (MMSE), 30 to 0 with death = −1

•Telephone-Mini Mental Status Examination (t-MMSE), 18 to 0 with death = −1

•Telephone Interview for Cognitive Status (TICS), 37 to 0 with death = −1

•Stroop (accuracy), 24 to 0 with death = −1

•Trail-making (accuracy) [46], 25 to 0 with death = −1

•Modified Rankin Scale (mRS), 0 to 5 with death = 6

•Barthel Index (BI), 100 to 0 with death = −5

•EuroQol EQ-5D Health Utility State (HUS), 1 to −0.594 with death = 0

•EuroQol Visual Analog Scale (EQ-VAS), 100 to 0 with death = −1.

•Zung Depression Scale (ZDS), 25 to 100 with death = 102.5

•Verbal fluency (animal naming), x to 0, with death = −1

#### Primary outcome

Comparison of ACE-R (extended to include death - section Missing data, and death) between ‘intensive’ and ‘guideline’ BP/lipid lowering groups using multiple linear regression and with adjustment (section Comparisons between treatment groups).

#### Secondary analyses

Dichotomous, ordered categorical, continuous and time to event data will be analysed using binary logistic regression (BLR), ordinal logistic regression (OLR), multiple linear regression (MLR) or Cox regression (CR) respectively, and with adjustment (section Comparisons between treatment groups). 95% confidence intervals will be given and *P* < 0.05 will be considered statistically significant.

The proportion of participants with cognitive impairment or who have died, and cognitive decline or died, will be compared between the treatment groups, as done previously for MMSE (a subset of ACE-R) [15,28]. Nevertheless, where possible, continuous or ordinal outcomes will be used in preference to dichotomous outcomes.

### Governance and funding

#### Trial Steering Committee (TSC)

The TSC provides leadership for the trial and determines and monitors the overall strategy. It meets annually, and has teleconference or Email discussions as needed.

#### Data Monitoring Committee (DMC)

The DMC reviews unblinded data annually in respect of safety and efficacy, and considers the study in the context of other trials of dementia prevention post-stroke. It meets at least annually.

#### Trial Management Committee (TMC)

The Trial Management Committee (TMC) runs the trial, with meetings every three weeks. It is unblinded to BP and lipid levels, and communicates with the Trial Steering Committee (TSC) and investigators as to whether targets are being met.

#### Adjudication committees

All adjudication is performed blinded to treatment assignment:

•Dementia is adjudicated by a group of three individuals (AB, CB, GF); each adjudicator sees each event

•Vascular events are adjudicated by a group of three individuals (PP, AM, RH); each adjudicator sees each event

•Serious adverse events are adjudicated by one of two adjudicators (NS, TE)

#### Sponsor

The University of Nottingham is the trial’s sponsor.

#### Funding

The start-up phase of PODCAST is funded jointly and equally by the UK Alzheimer’s Society and UK Stroke Association.

## Trial Status

A lower than planned recruitment of sites and patients has meant that the aspiration to recruit 600 patients over two years has not been realized. A number of reasons explain the poor recruitment:

•Research governance issues. The trial requires both acute hospital trusts (who identify patients, manage intensive BP and/or lipid lowering, and perform clinic follow-ups) and general practices, through Primary Care Trusts (PCTs, who manage guideline BP and/or lipid management), to sign-up. In general, each acute trust is associated with between one and three PCTs so that approval for each recruiting site requires between two and four agreements and contracts. It has proved difficult to coordinate the agreement to deliver the study in a locality between these acute and community trusts.

•NHS Excess Treatment Costs. PCTs were often unwilling to approve payment of treatment costs, often citing the cost of atorvastatin (which is now generic but was not so at the start of the trial).

•Long-term follow-up. Once a cohort of patients is recruited, each patient needs follow-up (which typically last two and a half hours) every six months, thereby placing a considerable work load on research staff at acute sites.

•Changes to the original protocol are summarised in Table 1

**Table 1:** Protocol amendments and other changes to trial practice

**Criterion**	**Previous versions**	**Current version/status**	**Reason**
**Protocol changes**			
Posterior circulation stroke (POCS)	Excluded	Included	To expand the inclusion criteria; posterior circulation stroke can lead to cognitive decline
Exclusion of NYHA 3 or 4	Exclusion criterion	Removed	To simplify protocol
LDL-c target	< 2.0 mmol/l	< 1.4 mmol/l	Half of patients already at LDL-c < 2 at baseline
Total cholesterol	< 4.0 mmol	< 3.1 mmol/l	Ditto
Glucose monitoring		Glucose, HbA1C	Some BP and lipid drugs may reduce, or cause, diabetes mellitus
Quality of life	DEMQOL	Removed	To simplify protocol
Screening	As telephone call	As research clinic visit	To reduce recruitment of ineligible patients
Time from screening to randomisation	2 weeks	1 week	To accelerate recruitment
Guideline statin dosage	Simvastatin 40 mg, pravastatin 40 mg, fluvastatin 40 mg	Simvastatin 10 to 40 mg, pravastatin 10 to 40 mg, fluvastatin 10 to 80 mg	To reflect NICE guidelines on lipid management (CG 67, 2008)
Statin classification	Guideline statin:	Guideline statin:	To clarify intensive versus guideline lipid lowering management
	simvastatin < 40 mg, pravastatin any dose, fluvastatin any dose, atorvastatin 10 mg.	simvastatin ≤ 40 mg, pravastatin any dose, fluvastatin any dose, atorvastatin ≤ 20 mg.	
	Intensive statin: atorvastatin ≤ 40 mg	Intensive statin: atorvastatin > 20 mg, rosuvastatin any dose	
Trial duration and participant involvement	8 years	4 years	To shorten trial since the main phase is no longer justified
BP and lipid management in follow-up visits		‘Floating’ visit at any time outside the planned visits at 3, 6, 12, 18, 24, 30, 36 and 42 months	To allow enhanced escalation of treatment, as appropriate
Baseline and follow-up BP and HR monitoring	Three measurements in rapid succession	Four measurements in rapid succession including one standing	To detect postural hypotension
Neuroimaging sub-study scan		CT scan on treatment (plus collection of any clinical scans during treatment)	To detect potential affects on atrophy, white matter changes
Follow-up visits	Seen in clinic once a year with interval blinded telephone follow-up.	Seen in clinic once every 6 months	To assess latest BP and/or lipid levels and escalate treatment as appropriate
Other changes			
Minimisation variables	As in section Minimisation (on key prognostic/logistical variables) above	Age, systolic BP, LDL-c	Small trial size precluded numerous minimisation variables
Email reminders		Twice yearly to investigators.	To highlight the need to achieve targets in BP and lipid lowering in patients randomised to intensive management

## Abbreviations

PODCAST: Prevention of Decline in Cognition after Stroke Trial; TICS: Telephone Interview for Cognitive Status; MOCA: Montreal Cognitive Assessment; ACE-R: Addenbrooke’s Cognitive Examintaion-revised; MMSE: Mini-Mental State Examination; od: once daily; bd: Twice daily; PCT: Primary Care Trust; IQCODE: Informant Questionnaire on Cognitive Decline in the Elderly; DSM IV: Diagnostic and Statistical Manual of Mental Disorders edition IV; BP: Blood pressure; SBP: Systolic Blood Pressure; DBP: Diastolic Blood Pressure; ABPM: Ambulatory Blood Pressure Monitoring; HR: Heart rate; MI: Myocardial infarction; PAD: Peripheral arterial disease; eGFR: estimated Glomerular Filtration Rate; ALT: ALanine Transaminase; IS: Ischaemic stroke; ICH: Intracerebral haemorrhage; SAE: Serious adverse events; GP: General practitioner; WMH: White matter hyperintensities; POCS: Posterior Circulation Stroke; ARR: Absolute risk reduction; NNT: Number-needed-to-treat; RRR: Relative risk reduction; ITT: Intention-to-treat; CT: Computed Tomography; MR: Magnetic Resonance imaging; TC: Total cholesterol; TG: Triglycerides; HDL-c: High Density Lipoprotein cholesterol; LDL-c: Low Density Lipoprotein cholesterol; OAST: Optimizing Analysis of Stroke Trials’; EMEA: European Agency for the Evaluation of Medicinal Products; BLR: Binary logistic regression; OLR: Ordinal logistic regression; MLR: Multiple linear regression; CR: Cox regression; mRS: Modified Rankin Scale; BI: Barthel Index; HUS: EuroQol EQ-5D Health Utility State; ZDS: Zung Depression Scale; TMC: Trial Management Committee; TSC: Trial Steering Committee; DMC: Data Monitoring Committee.

## Competing interests

The authors declare that they have no competing interests.

## Authors’ contributions

DJB and PB drafted the manuscript and updated the current literature for the background section. Statistical advice was provided by SP. CB, PB, AB, GF, JM, PP, SP, JR, RS and JW designed the study and wrote the protocol. All authors read and improved the final manuscript.
